# The Time Preferred by Patients to Undergo Surgery: What Proportion Would Accept Day-Case Versus Overnight Tonsillectomy/Adenotonsillectomy?

**DOI:** 10.24248/eahrj.v7i1.717

**Published:** 2023-07-12

**Authors:** Zephania Saitabau Abraham, Aveline Aloyce Kahinga

**Affiliations:** a Department of Surgery, School of Medicine and Dentistry, University of Dodoma, Dodoma, Tanzania; b Department of Otorhinolaryngology, Muhimbili University of Health and Allied Sciences, Dar es Salaam, Tanzania

## Abstract

**Background:**

Day-case tonsillectomy is becoming popular with varying patients' satisfaction. Whether day-case or overnight tonsillectomy is safe and preferred by patients has been debatable among otorhinolaryngologists. To date, majority of otorhinolaryngologists in Tanzania are practicing overnight tonsillectomy unlike what is being practiced in other parts of the world. There is scarce literature regarding whether day-case or overnight surgical option is preferred by patients.

**Objective:**

To assess the time preferred by patients to undergo surgery by ascertaining the proportion of patients who would prefer day-case to overnight tonsillectomy or adenotonsillectomy.

**Methods:**

We conducted a hospital based cross sectional study at Ekenywa Specialised Hospital from January to December 2021. The study recruited 200 patients who underwent elective tonsillectomy or adenotonsillectomy. A structured questionnaire adopted from previously published studies and thereafter modified accordingly to fit the current study was used to collect relevant data. Data were analysed using Statistical Package for Social Sciences (SPSS) version 21

**Results:**

The study had more male participants 104(52%) than females, 96 (48%) with a male to female ratio of 1.1:1. Majority of the study participants were aged below 5 years. Male participants aged below 5 years accounted for 50% while females aged <5 years accounted for 65.6%. Of the 200 (100%) patients who returned the questionnaires, 135(67.5%) preferred discharge on the 1^st^ day post surgery while 65 (32.5%) preferred discharge on the 2^nd^ postoperative day or later. Similarly, five (2.5%) would have wished day-case tonsillectomy/adenotonsillectomy while 195(97.5%) preferred an overnight surgery. Patients with postoperative pyrexia, older patients and those discharged on the 2^nd^ day post surgery or later were more likely to prefer a longer postoperative hospital stay.

**Conclusion:**

Day-case surgery seems feasible due to less risk of postoperative complications such as haemorrhage and fever. Majority of the participants in this study preferred overnight tonsillectomy/adenotonsillectomy.

## INTRODUCTION

Day-case tonsillectomy/adenotonsillectomy is becoming increasingly popular worldwide. Published studies elsewhere are reporting day-case tonsillectomy/adenotonsillectomy to be safe in both adults and children.^1,2^ However, observations carried out in various public and private health facilities in Tanzania report that most otorhinolaryngologists practice overnight tonsillectomies and adenotonsillectomies. Although patient selection in day-case surgery is based on strict and relatively uniform criteria, patient satisfaction with same-day discharge tend to vary. In a study conducted in Portugal, overall satisfaction following day surgery was at least 95% at discharge and at 30 days. However, complete satisfaction was present only in 75% at discharge and it reduced to 62% at 30 days.^3^ Controversies on whether to adopt day-case or overnight tonsillectomy/adenotonsillectomy have necessitated studies to determine acceptability and safety of the two. A study conducted in Jordan involving 419 paediatric patients, 103 inpatients and 316 day-case group reported that only one patient (0.32%) among the day-case patients had secondary post-tonsillectomy bleeding. Similarly, there was only one case of this reported for the inpatient group (1%), and with none reactionary hemorrhage reported in either groups. The study found no significant association between the inpatient and day-case surgery groups in terms of post-tonsillectomy bleeding.^4^ Another study conducted in England to assess the safety of day-case paediatric tonsillectomy found the rates of adverse postoperative outcomes to be similar for those who discharged >70% children the day-case post tonsillectomy compared with those who discharged <50% children the day-case post tonsillectomy and therefore no consistent evidence that day-case tonsillectomy is associated with poor outcomes.^5^ A study conducted in France to audit changes in practice following the opening up of a day-surgery unit with dedicated operating room found that the opening of the day-surgery unit led to changes in practice, with most tonsillectomies being performed on an outpatient basis, without increased complications, and notably immediate complications.^6^ Similarly, day-case tonsillectomy in children showed a 30-day complications rate comparable to those reported in the literature (8.3% postoperative hemorrhage), with a higher rate in adults (35.3%).^6^ A systematic review of outpatient tonsillectomy in children aiming at determining the level of evidence regarding the safety of outpatient paediatric tonsillectomy having pooled 17 articles found that the current level of evidence supports the practice of outpatient tonsillectomy in properly selected children.^7^

A study that was conducted in Finland to determine patients' preferences for length of hospital stay post adenotonsillectomy found 39.8% of the study participants to have preferred discharge on the first day, while 59.7% preferred discharge on the second postoperative day or later, and only one patient (0.4%) wished for day-case tonsillectomy.^2^

There is scarce literature on the same topic in Africa. A study conducted in Tanzania on the survey of tonsillectomy care patterns reported 34.6% of the otorhinolaryngologists to have never performed day-case tonsillectomy while 65.4% sometimes performed day-case tonsillectomy.^8^ Whether the day-case tonsillectomy and adenotonsillectomy being reported by otorhinolaryngologists is preferred by patients or not, has not been assessed. Our study aims to address this existing knowledge gap.

To address this gap, this study evaluated patients undergoing elective tonsillectomy and adenotonsillectomy over 12 months and their opinion as to whether they would prefer day case or overnight tonsillectomy/adenotonsillectomy. This is the first study in our country to assess the time preferred by patients to undergo the mentioned surgery with emphasis on what proportion would accept day-case or overnight tonsillectomy/adenotonsillectomy.

## METHODS

### Study Area and Population

The study was conducted at Ekenywa Specialised Hospital, Department of Otorhinolaryngology from January to December 2021. The study recruited 200 patients that were undergoing elective tonsillectomy or adenotonsillectomy. The department of otorhinolaryngology attends to about 200 outpatients on daily basis and performs an average of 6 elective surgeries per day.

### Study Design

A hospital based cross sectional study was conducted to determine the time preferred by patients to undergo tonsillectomy/adenotonsillectomy by ascertaining the proportion of patients who prefer a day-case or an overnight option for the scheduled surgery.

### Inclusion Criteria

Healthy patients who underwent elective bilateral tonsillectomy with or without adenoidectomy upon obtaining their consent to participate were included in the study.

### Exclusion Criteria

Patients who underwent elective bilateral tonsillectomy with or without adenoidectomy and were unwilling to participate were excluded. Also patients who were mentally unfit to consent to participate were also excluded.

### Sample Size and Sampling Technique

Data was collected using convenient sampling technique. Study participants were recruited on convenient basis until the desired sample size was achieved. Moreover, the sample size for this study was calculated using the formula below;



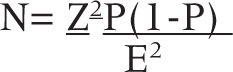



Where;

n = sample size

z= standard normal deviate=1.96 for 95% confidence level

p = proportion, P=75.4%, proportion of day-case tonsillectomy/adenotonsillectomy.^4^

E= margin of error which is 5%

Therefore, N=1.96^2^ ×75.4(100 75.4)/5^2^ = 285

The minimum estimated sample size was 285 study participants; however, only 200 participants were recruited.

### Data Collection Tool

A structured questionnaire adopted from previously published studies and thereafter modified accordingly to fit the current study's goal and objectives was used to collect data.^2,6,9^ The first version of the questionnaire was prepared in English and the final draft was translated to Swahili since the study participants attending the health facility were more conversant with Kiswahili. The questionnaire comprised of 2 main parts with sub-sections: (i) Age and sex distribution of the study participants (ii) Clinical profile of the study participants and this included; (a) Indications for tonsillectomy/adenotonsillectomy (b) Discharge summary of the study participants particularly the specific day of discharge post surgery (whether first day or second day post surgery) (c) their preference on when they should be discharged post surgery (d) Postoperative outcomes such as postoperative pyrexia, postoperative haemorrhage. The questionnaires comprised of closed ended questions and were self-administered. The procedure included self-introduction by the principal researcher, introduction of the topic and purpose of the study. Participants were assured of their free participation and withdrawal will from the study at any time if they wish to do so. Tool validity was assessed by reviewing the literature as well as pilot testing the instrument prior to the study by involving 10% of the actual sample size from the specialised hospital and these were excluded from the actual study.

### Recruitment Strategy

Patients were mandated to report at the hospital one (1) day before surgery and on admission each patient received details of the anticipated procedure including the course of recovery and possible postoperative complications and for those under the age of 18 years, same information was conveyed to them and to their respective parents/caretakers. Tonsillectomy was performed under general anaesthesia with a bipolar electro dissection technique.

An otorhinolaryngologist at the ear, nose and throat (ENT) department discharged all patients at the earliest 1^st^ day post surgery and provided them with verbal information on the course of recovery. Prolonged hospital stay was considered if the patient had a medical reason such as post-tonsillectomy haemorrhage or pyrexia (body temperature higher than 37.5 degree Celsius). Each patient received a prescription for non-steroidal anti-inflammatory analgesic and an oral antibiotic post tonsillectomy/adenotonsillectomy and for those who underwent adenotonsillectomy, were prescribed with a nasal decongestant (ephedrine nasal drops). All patients and, in the case of those under the age of 18 years had their parents/caretakers providing a written informed consent for the planned surgery.

### Statistical Analysis

The collected data were cleaned and analysed using SPSS version 21 software package. Descriptive statistics were performed to present frequency distribution for demographic characteristics and clinical profile of the study participants.

Chi-square test was performed to establish the relationship between the selected independent and dependent variables. All the independent variables with p-value <.05 were regarded to be statistically significant. Statistical limits were set at a 95% confidence interval and a 5% significance level.

### Ethical Considerations

A written informed consent was obtained from all participants. For participants below 18 years, written informed consent was obtained from their respective parents/guardians. The aim of the study was explained to patients, parents and caretakers. Ethical approval from the hospital ethics committee was granted on 10^th^ December 2020 with number Ref: *ESH/2020/12*. Participants were assured of their privacy and confidentiality. Anonymity was maintained by the use of code number on the questionnaire instead of the participant's name and the participant had an absolute freedom and right to withdraw from the study at any time without any compromise of the medical care they were receiving at the hospital.

## RESULTS

### Age and sex distribution of study participants, discharge pattern from the hospital and postoperative outcomes after elective tonsillectomy/adenotonsillectomy

In this study, there were more males, 104(52%) than female, 96 (48%) participants. Male to female ratio was recorded at 1.1:1. Majority of the study participants were aged below 5 years, where males aged below 5 years accounted for 50% while females aged below 5 years accounted for 65.6% of the respective gender. (Table 1)

**TABLE 1:** Distribution of Study Participants According to Age and Sex

Sex	Age groups (years) (%)							Total (%)
	<5 (%)	6–10 (%)	11–15 (%)	16–20 (%)	21–25 (%)	26–30 (%)	31+	
Male	52 (50.0)	26 (25.0)	11 (10.6)	9 (8.7)	1 (0.9)	3 (2.9)	2 (1.9)	104 (52)
Female	63 (65.6)	7 (7.3)	11 (11.4)	4 (4.2)	3 (3.1)	4 (4.2)	4 (4.2)	96 (48)
Total	115 (57.5)	33 (16.5)	22 (11)	13 (6.5)	4 (2)	7 (3.5)	6 (3)	200 (100)

On the other hand, of all the 200 surgical patients, 188 (94%) patients underwent adenotonsillectomy but 12(6%) underwent tonsillectomy. The indication was obstructive tonsillitis in 6 patients (50%), recurrent acute tonsillitis in 2 (16.7%), and chronic tonsillitis in 4 (33.3%). Obstructive adenotonsillitis was the commonest indication in younger age groups and obstructive tonsillitis in those aged 11+ years. Majority of patients across all age groups in this study were discharged on the first day postoperatively. 1 patient (0.5%) had primary post-tonsillectomy hemorrhage and 2 patients (1%) had primary post-adenotonsillectomy hemorrhage and all the 3 patients were aged <5years. Similarly, 3 patients had secondary post adenotonsillectomy hemorrhage (1.5%). No hemorrhage post adenoidectomy was encountered in this study. Postoperative fever was encountered in 7(3.5%) patients and all were aged <5 years. (Table 2)

**TABLE 2:** Age of Study Participants and Discharge Pattern from the Hospital after Elective Tonsillectomy/Adenotonsillectomy

Age (years)	Discharge from the hospital, n (%)		Total n (%)
	1st postoperative day	2nd postoperative day	
<5	105 (91.3	10 (8.7)	115 (100)
6–10	30 (90.9)	3 (9.1)	33 (100)
11–15	15 (68.2)	7 (31.8)	22 (100)
16–20	12 (92.3)	1 (7.7)	13 (100)
21–25	3 (75)	1 (25)	4 (100)
26–30	5 (71.4)	2 (28.6)	7 (100)
31+	5 (83.3)	1 (16.7)	6 (100)
Total, n (%)	175 (87.5)	25 (12.5)	200 (100)

Regarding patients' discharge preference after elective surgery, majority 135(67.5%) preferred discharge to happen 1^st^ day after surgery. Similarly, those patients who had 2 nights hospital stay post surgery and those with postoperative fever preferred discharge to happen longer (2^nd^ day or thereafter post surgery) (*p-value=.001*). (Table 3)

**TABLE 3:** Patients' Discharge Preference after

When discharge should have occurred after surgery	Frequency, n (%)
1st day postoperative	135 (67.5)
2nd day postoperative or longer	65 (32.5)
Total, n (%)	200 (100)

## DISCUSSION

Tonsillectomy being one of the commonest surgery in otorhinolaryngology has been performed invariably in different parts of the world with other surgeons preferring patients to be admitted a day before surgery (overnight) and other being admitted on the same day when surgery has to be performed. Whether patients prefer day-case tonsillectomy/adenotonsillectomy or an overnight surgical option remains to be unknown in Tanzania and this study aimed at addressing such gap. Regarding patients' views on whether they would prefer overnight or day-case tonsillectomy, majority (97.5%) reported preference of overnight surgery and thus day-case surgery to patients was found to be undesirable.

This finding appears to be similar to what was established in a study that was conducted in Finland. ^2^ On the other hand, since our patients had no systemic illnesses (ASA class 1), majority of them would have been suitable for day-case surgery but because in the private health facility where the study was conducted day-case surgery was not an option to be considered but rather all patients have to be admitted a day before adenotonsillectomy/tonsillectomy and this might have probably influenced patients' attitudes on discharge preference. Patients' acceptability of day-case adenotonsillectomy is higher in a study that was conducted in a day-surgery unit in the United States ^10^, than involving in-patient operations in a study that was conducted in the United Kingdom. ^11^

Regarding patients' discharge preference after elective surgery, majority, (67.5%) preferred discharge to happen 1^st^ day after surgery. Such finding appear to correlate with what was found in studies done elsewhere. ^2,12^ In our study, those patients who had 2 nights hospital stay post surgery and those with postoperative fever preferred discharge to happen longer (2^nd^ day or thereafter post surgery) (p-value=0.001)

Pertaining patients' and suitability for day-case adenotonsillectomy, our study found children aged <5 years unsuitable for day-case surgery since both children who suffered from postoperative hemorrhage were aged <5years and similarly all the seven children who developed postoperative fever were aged <5 years. Such findings appears to be dissimilar to those found in a study which was conducted in Finland where children younger than 10 years seemed to be the best group for day-case tonsillectomy because they were the least likely to suffer from postoperative fever or primary postoperative hemorrhage. ^2^

Regarding whether day-case adenotonsillectomy/tonsillectomy should be adopted or not from our findings where majority of the study participants found overnight surgery to be the preferred choice should be taken with caution since these are findings from a single health facility and therefore multi-centered studies should be conducted on the same topic and this must be coupled with adequate preparations if at all day-case surgery has to be adopted in our country. Preparations should include preparedness of health workers in accepting the new treatment policy and also make meticulous preoperative preparations to patients' who are to undergo day-case adenotonsillectomy/tonsillectomy. Pilot health facilities to perform day-case surgery may be designed countrywide to check feasibility of such surgical strategy

## CONCLUSION

Overnight tonsillectomy/adenotonsillectomy has been the preferred surgery in this study and on the other hand day case tonsillectomy/adenotonsillectomy from our findings seems feasible due to less risk of postoperative complications such as postoperative hemorrhage and fever. Large multicentric studies should be designed to validate these findings and ascertain on the possibility of implementing day-case surgery in our settings.

### Study Limitations

The study was conducted in a single health facility and considered a limited sample size. Thus, the results from the study cannot be generalised. Larger multicentric studies incorporating relevant socio-demographic information like residence, level of education and profession should be designed so as to assess their influence on patients' discharge and type of surgery opted for.
